# A Novel Approach to Identify Sundowning with Cyclomatic Complexity Movement Analysis and Noninvasive Ambient Sensors

**DOI:** 10.1002/alz.091052

**Published:** 2025-01-09

**Authors:** Brian B Johnson, Radhakrishna S Jamadagni, Ida Bagus Kresnasandi, Britni A Bolstad

**Affiliations:** ^1^ University of Minnesota, Minneapolis, MN USA; ^2^ WellBe Senior Medical, Atlanta, GA USA

## Abstract

**Background:**

Sundowning is the development or progression of neuropsychiatric symptoms (NPS) often occurring in the afternoon or early evening. Noninvasive ambient sensors (NAS) monitor individuals without the need to wear a device or use a camera. The data from NAS sensors can identify movement patterns in the context of cyclomatic complexity to indicate when an individual may be sundowning. This approach could improve the quality of life (QOL) for individuals with Alzheimer’s disease (AD) and their caregivers.

**Method:**

NAS sensors placed in an individual’s home could identify wandering due to sundowning when new patterns of movement emerge. Quantification of movement with cyclomatic complexity uses machine learning (ML) to identify and notify caregivers when new sequences of room transitions emerge. Cyclomatic complexity is represented as M = E − N + 2P where E is the amount of walking paths, N is the number of rooms, P is the total number of connected rooms (Khan & Jacobs). A new pattern of movement categorized as wandering is one signal a caregiver could consider within the constellation of symptoms.

**Result:**

The method of approaching quantification of movement in an ML framework was pioneered by Dr. Taha Khan and Dr. Peter G. Jacobs in the study “Prediction of Mild Cognitive Impairment Using Movement Complexity”. Labeling sequences of human movement within an ML framework could lead to novel insights for understanding human behavior. Individuals that prefer to age‐in‐place could benefit from early detection of neuropsychiatric symptoms and prevention of sentinel events with proactive clinical decision‐making.

**Conclusion:**

The transition from CIN to MCI is a significant change for an individual. Current assessments are dependent on the clinical interpretation by the caregiver. Instead of waiting until a sentinel event occurs, predictive algorithms could be used for early intervention. Data could enhance the decision making‐support for further assessment or admission to a long‐term care facility. As the aging population continues to grow, affordable technological solutions will be critical for monitoring health factors in real‐time. By providing this early warning, caregivers can adjust their routines and interventions to better support the patient.

